# Circulating miR-141 as a potential biomarker for diagnosis, prognosis and therapeutic targets in gallbladder cancer

**DOI:** 10.1038/s41598-022-13430-8

**Published:** 2022-06-16

**Authors:** Ganghua Yang, Zhengyang Lu, Fandi Meng, Yong Wan, Lei Zhang, Qinhong Xu, Zheng Wang

**Affiliations:** 1 Department of Geriatric Surgery, First Affiliated Hospital of Xi’an Jiaotong University, Xi’an, 710061 Shaanxi Province China; 2 Department of Hepatobiliary Surgery, First Affiliated Hospital of Xi’an Jiaotong University, 277 West Yanta Road, Xi’an, 710061 Shaanxi Province China

**Keywords:** Cancer, Cancer therapy, Gastrointestinal cancer, Tumour biomarkers

## Abstract

MicroRNA-141(miR-141) has been reported to play vital roles in the regulation of carcinogenesis and cancer progression. However, the biological function of miR-141 in GBC has received less attention. The aim of this study was to estimate the potential value of the expression level of miR-141 as a diagnostic and prognostic blood-based biomarker in gallbladder cancer (GBC) patients. Meanwhile, to explore its biological role in GBC cells. RT-PCR was employed to confirm the expression of miR-141 in ten paired tissue samples (10 GBC tissues and 10 adjacent normal gallbladder tissues), GBC cell lines and peripheral blood specimens from 98 GBC patients and 60 healthy controls. MTT assay was used to evaluate the GBC cells proliferation and flow cytometry was used to detect the cell apoptosis. Receiver operating characteristic curve analysis and the area under the curve (AUC) were used to evaluate the value of miR-141 plasma levels for GBC diagnosis. Finally, clinicopathological and survival data of all GBC patients were collected and analyzed. Here, we confirmed that the expression of miR-141 were upregulated in primary gallbladder cancer cells and tissues compared with human gallbladder epithelial cells and adjacent normal tissues (*P* < 0.0001). Meanwhile, we found that downregulated expression of miR-141 by miR-141 inhibitor could induce apoptosis and inhibit proliferation of GBC cells. Additionally, elevated plasma miR-141 expression was also detected in the peripheral blood of GBC patients compared with healthy controls (*P* < 0.0001). The AUC value of miR-141 for GBC diagnosis was 0.894 (95% CI 0.843–0.945), which was more valuable than those including carcinoembryonic antigen (CEA) (0.713, 95% CI 0.633–0.793), carbohydrate antigen 125 (CA125) (0.837, 95% CI 0.776–0.899) and carbohydrate antigen 19–9 (CA19-9) (0.869, 95% CI 0.813–0.924). The high expression level of miR-141 in plasma was significantly associated with tumor invasion (*P* = 0.008), lymph node metastasis (*P* < 0.0001) and advanced pathologic tumor/node/metastasis (pTNM) stage (*P* = 0.009). More importantly, high plasma miR-141 expression was an independent prognostic factor for predicting poorer long-term survival in GBC patients. Elevated expression of circulating miR-141 in peripheral blood might be a potential novel biomarker for diagnosis and prognosis of GBC patients. Downregulated expression of miR-141 could inhibit proliferation and induce apoptosis of GBC cells, that provide a potential therapeutic target for GBC.

## Introduction

Gallbladder cancer (GBC) is the most common aggressive biliary tract malignancies and has a poor prognosis. There were 219,420 new GBC cases and 165,087 GBC deaths worldwide in 2018^1^. The 5-year survival rate of GBC patients is less than 10%^2–5^. Even after undergoing radical cholecystectomy, neoadjuvant or adjuvant chemotherapy, most of GBC patients eventually experience local recurrence or distant metastasis. Early diagnosis is essential for improving the prognosis and long-term survival of GBC patients. However, there is no specific biomarker for the early diagnosis and prediction of the clinical outcome for patients with GBC. Hence, the development of novel molecular markers that can predict prognosis and be helpful for early diagnosis is urgently needed.

MicroRNAs (miRNAs, miRs) are single-stranded non-coding RNA molecules constituting of approximately 18–25 nucleotides that negatively regulate target genes by translational repression or the degradation of complementary mRNAs. MiRNAs have been reported to play vital roles in the regulation of carcinogenesis and cancer progression^6–8^. Recent studies have demonstrated that tumor‑related miRNAs were appear in patient’s peripheral blood with remarkably stable status that can resistant to degradation of endogenous ribonuclease. The aberrant expression of miRNAs in plasma may be advantageous for early diagnosis and predicting the survival of patients and is expected to become a potential valuable biomarker for the clinical assessment of cancers^9–12^.

MicroRNA-141 (miR-141) has been reported to play an important role in carcinogenesis and regulating the development and progression of cancer^13,14^. According to previous research, miR-141 possessed tumor suppressive or oncogenic activities in various human malignancies. For instance, Li et al.^15^ demonstrated that the overexpression of miR-141 in gastric cancer cells could suppress cell proliferation and apoptosis. Similarly, Long et al.^16^ found that miR-141 functions as a tumor suppressor to inhibit the proliferation and migration of colorectal cancer cells. In contrast, a few previous studies found that the overexpression of miR-141 in prostate cancer and esophageal cancer could increase the risk of local recurrent or distant metastasis and presented worse survival^17,18^. However, the biological function of miR-141 in GBC has attracted less attention. A recent study demonstrated that the overexpression of miR-141 was significantly associated with shorter disease-free survival (DFS), poorer overall survival (OS) and a greater risk of angiolymphatic invasion in patients with biliary tract cancer^19^. More important, a part recent research have shown that serum miR-141 can be used as a novel biomarker for the diagnosis and prognosis of cancers^20,21^. Thus, we hypothesized that the expression of circulating miR-141 in plasma could be a novel and useful predictive biomarker for diagnosis, therapy and prognosis in GBC patients.

The aims of this study were to evaluate the expression level of circulating miR-141 in peripheral blood of GBC patients and healthy volunteers to confirm whether a correlation exists between the expression of miR-141 and the clinical outcomes of GBC. We also compared the diagnostic specificity and sensitivity of circulating miR-141 in plasma with those of conventional GBC biomarkers, such as carcinoembryonic antigen (CEA), carbohydrate antigen 125 (CA125) and carbohydrate antigen 19–9 (CA19-9) in serum. In addition, we estimated the biological roles of miR-141 on GBC cells proliferation and apoptosis.

## Materials and methods

### Study population

A total of 98 blood samples from consecutive GBC patients (mean age 57.8 ± 9.6 years; range 30–76 years) were collected before resection at the First Affiliated Hospital of Xi’an Jiaotong University from January 2017 to January 2020. All GBC cases were confirmed by histopathology in patients who underwent R0 or R1 resection, and no chemotherapy treatments were given before surgery. As a control, blood samples from 60 healthy participants (mean age 56.5 ± 6.9 years; range 36–70 years) without any cancerous disease were collected at the Physical Health Examination Center of the First Affiliated Hospital of Xi’an Jiaotong University. Peripheral blood (5 ml) was gathered in EDTA-K2 anticoagulant tubes from the healthy controls and from each GBC patient at the time before the surgery. The blood specimens were immediately underwent two-spin protocol (3000 rpm for 20 min and 12,000 rpm for 10 min at 4 °C) to prevent contamination by cellular nucleic acids, and then, stored at – 80 °C for further analysis after the total RNA was extracted. Additionally, ten paired tissue samples (10 GBC tissues and 10 adjacent normal gallbladder tissues) were collected at the time of surgery and stored at – 80 °C for further analysis. Tumor stage was assessed according to the eighth edition of the criteria established by the International Union Against Cancer (UICC) in 2017. Written informed consent was obtained from each participant before the blood sample collection. All procedures performed in experiments involving tissue and blood of human participants were in accordance with the ethical standards of the institutional and/or national research committee and with the Helsinki Declaration of 1975, as revised in 1983.

### Cell lines and cell culture

Human GBC cell lines (GBC-SD cells and SGC996 cells) and the human normal biliary epithelial cell line (H69) were purchased from the Shanghai Institute for Biological Science, Chinese Academy of Science (Shanghai, China). NOZ and OCUG-1 GBC cells were purchased from the Health Science Research Resources Bank (Osaka, Japan). GBC-SD, OCUG-1 and H69 were cultured in high-glucose DMEM (Gibco; Thermo Fisher Scientific, Inc., Waltham, MA, USA)). NOZ was cultured in Williams’ E medium (Invitrogen; Thermo Fisher Scientific, Inc.). SGC-996 cells were cultured in RPMI 1640 medium (Gibco; Thermo Fisher Scientific, Inc.). All types of culture medium were supplemented with 10% fetal bovine serum (Gibco; Thermo Fisher Scientific, Inc.) and 1% penicillin–streptomycin (Gibco; Thermo Fisher Scientific, Inc.) and maintained at 37 °C in a humidified atmosphere with 5% CO_2_*.*

### RNA extraction and quantitative RT-PCR

Total RNA was extracted using TRIzol Reagent (Invitrogen, Carlsbad, CA, USA) from plasma, tissues and cultured cells. The reverse transcription reaction was carried out using the Prime Script II cDNA Synthesis Kit (Takara Biotechnology Co., Ltd., Dalian, China) according to the manufacturer’s protocol. Real-time PCR was carried out in a 20 μl reaction mixture consist of 2 μl of cDNA template (200 ng), 10 μl of 2 × SYBR Green Mix, 0.5 μl of 200 nM forward and reverse primers, and 6 μl of nuclease-free water. Real-time PCR of miRNA was carried out using a 7500 Real-time PCR system (Applied Biosystems; Thermo Fisher Scientific, Inc.) by using the miDETECT A TrackTM miRNA qRT-PCR Kit (RiboBio Co., Ltd., Guangzhou, China) in accordance with the manufacturer’s protocol, and RNU6B (U6 control) was used as a housekeeping control. The forward primers for miR-141 and RNU6B were synthesized by the Shanghai Sangon Biotechnology Co. Ltd. (Shanghai, China). All primer sequences were as follows: miR-141 forward, 5′-ACACTCAGGTAGAAATG-3′ and reverse, 5′-ATTGTGACACCGAGTAC-3′; U6 forward, 5′-CTCGCTTCGGCAGCACA-3′ and reverse, 5′-ACGCTTCACGAATTTGC-3′. The expression of miR-141 from the samples was normalized to U6, and the relative expression of miR-141 was measured using the 2^−ΔΔCt^ method. ΔCt was estimated by subtracting the Ct values of U6 from that of the miRNA. ΔΔCt was then calculated by subtracting the ΔCt of normal samples from the ΔCt of GBC samples. The change in gene expression was estimated using the equation 2^−ΔΔCt^. Each sample was tested in triplicate.

### Transient transfection

miR-141 inhibitor and miR control inhibitor were purchased from Shanghai GenePharma Co., Ltd. (Shanghai, china). Cultured GBC-SD cells and SGC996 cells were selected and transfected with the miR-141 inhibitor or miR control inhibitor using the Lipofectamine 2000 transfection kit (Invitrogen Life Technologies, Carlsbad, CA, USA) in accordance with the manufacturer's protocol. Untransfected GBC cells served as the mock control. Transfections were performed in duplicates and repeated at least twice in each experiment.

### MTT proliferation assay

For the MTT assay, the GBC-SD cells and SGC996 cells transfected with the miR-141 inhibitor or miR control inhibitor were added to a 96-well plate. In order to evaluate the proliferation of the transfected GBC cell lines, 20 µl MTT assay (0.5 mg/ml; Sigma, St. Louis, MO, USA) solution was added to each well for 24 h, 48 h, 72 h, and 96 h respectively. Untransfected GBC cells served as the mock control.

### Cell apoptosis assay

The GBC-SD cells and SGC996 cells were transfected with the miR-141 inhibitor or miR control inhibitor and added to each well of a 96-well plate for 48 h. After that, the cells were collected and dyed with an Annexin V-fluorescein isothiocyanate (FITC) and propidium iodide (PI) (Roche, Mannheim, Germany) at room temperature for 15 min in the dark following with the manufacturer's protocols. Then, the cell apoptosis was detected with flow cytometry.

### Conventional tumor biomarker detection

The Cobas E-602 analyzer (Roche, Basel, Switzerland) was used to analyze the levels of serum CEA, CA125 and CA19-9 in 98 GBC patients and 60 healthy volunteers based on electrochemiluminescence (ECL).

### Survival data from gallbladder cancer patients

All of the GBC patients were followed up regularly every 3–6 months by our clinicians for more than 50 months or until death. The OS period was defined as the interval from the date of the initial diagnosis of GBC to death or the last follow-up. The last follow-up assessments were conducted in January 2021.

### Statistical analysis

Statistical analyses were performed using SPSS, version 19.0 (IBM, Chicago, IL, USA) and Graph Pad Prism version 5.0 (Graph Pad Software San Diego, CA, USA). Data are expressed as the means ± SD. One-way ANOVA and unpaired Student's t-test were performed to evaluate statistical differences. The chi-squared test was used to analyze the correlations between the expression levels of plasma miRNA-141 and the clinicopathological features. The receiver operating characteristic (ROC) curve analysis was used to assess the GBC related markers (circulating miRNA-141, serum CEA, CA125 and CA19-9); and the sensitivity, specificity, and area under the curve (AUC) values were determined. A univariate analysis of OS was evaluated by the Kaplan–Meier method and compared with the log-rank test. The Cox proportional hazards regression analysis was carried out to evaluate the independent prognostic factors that affect GBC patient survival. A *P* value < 0.05 was considered statistically significant.

### Institutional review board statement

This study was reviewed and approved by Ethical Review Committee of the First Affiliated Hospital of Xi’an Jiaotong University.

## Results

### The expression levels of miRNA-141 were upregulated in primary gallbladder cancer cells and tissues

**Figure 1:**
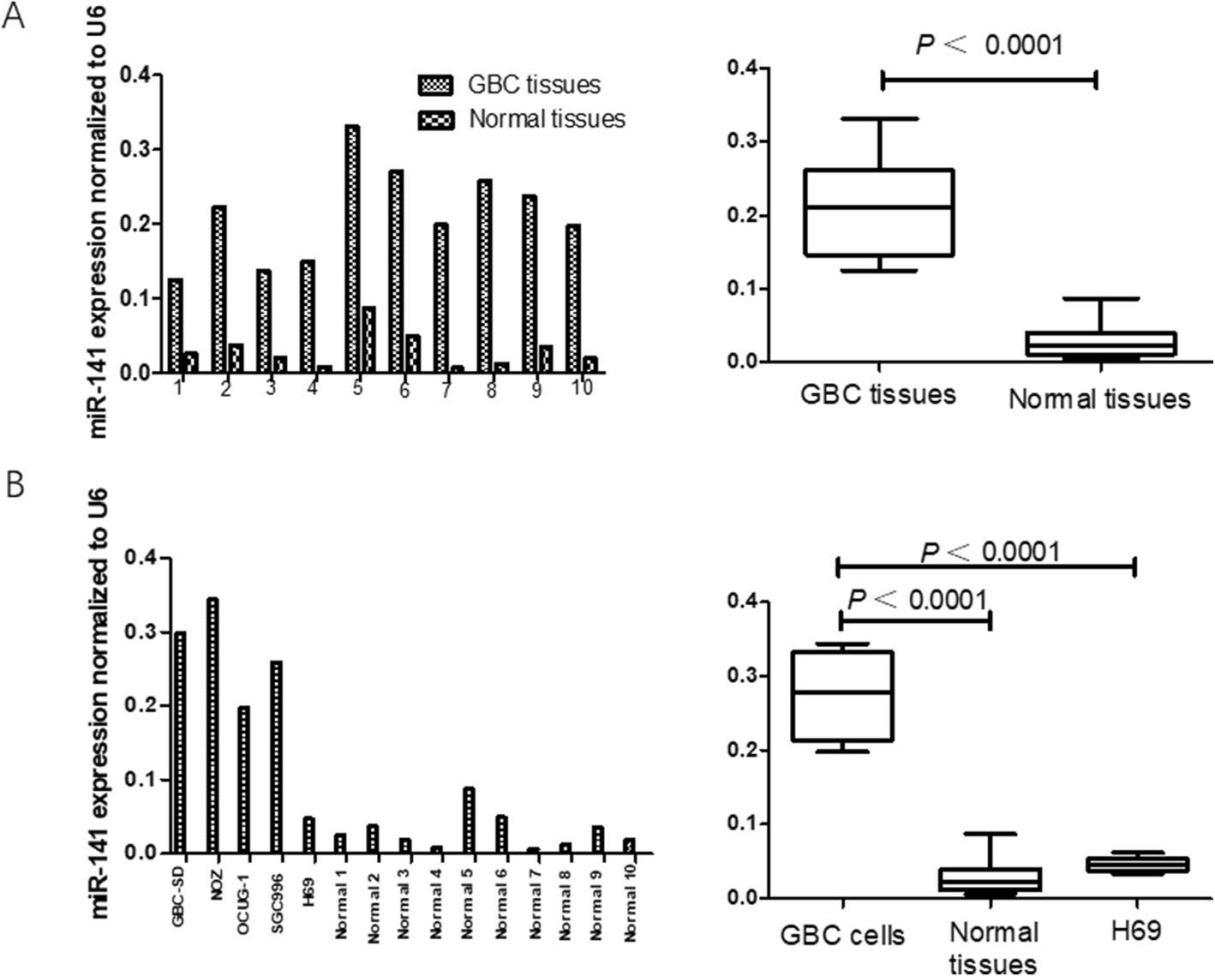
The expression levels of miR-141 in gallbladder cancer (GBC) tissues and GBC cell lines. (**A**) The expression levels of miR-141 were significantly higher in GBC tissues than in paired adjacent normal gallbladder tissues (*P* < 0.0001; Wilcoxon t-test). (**B**) The expression levels of miR-141 were significantly higher in GBC cell lines than in the human normal biliary epithelial cell line (H69) and normal tissues (*P* < 0.0001; Mann–Whitney U-test). RNU6B (U6) was used as an internal control.

To confirm the overexpression of miRNA-141 in GBC in previous studies, real-time PCR was used to analyze the expression of miRNA-141 in 10 paired GBC tissues and adjacent normal tissues (Fig. 1A). Additionally, the expression levels of miR-141 in 4 human GBC cell lines (GBC-SD, NOZ, OCUG-1, and SGC996) and the human normal biliary epithelial cell line H69 were also detected by RT-PCR (Fig. 1B). As shown in Fig. 1, the expression level of miRNA-141 in the examined GBC cell lines was remarkably higher than that in the H69 and normal tissues (*P* < 0.0001). Additionally, the miR-141 expression levels were significantly higher in GBC tissues than in paired adjacent normal gallbladder tissues (*P* < 0.0001).

### The overexpression of miRNA-141 was detected in the plasma of GBC patients

**Figure 2:**
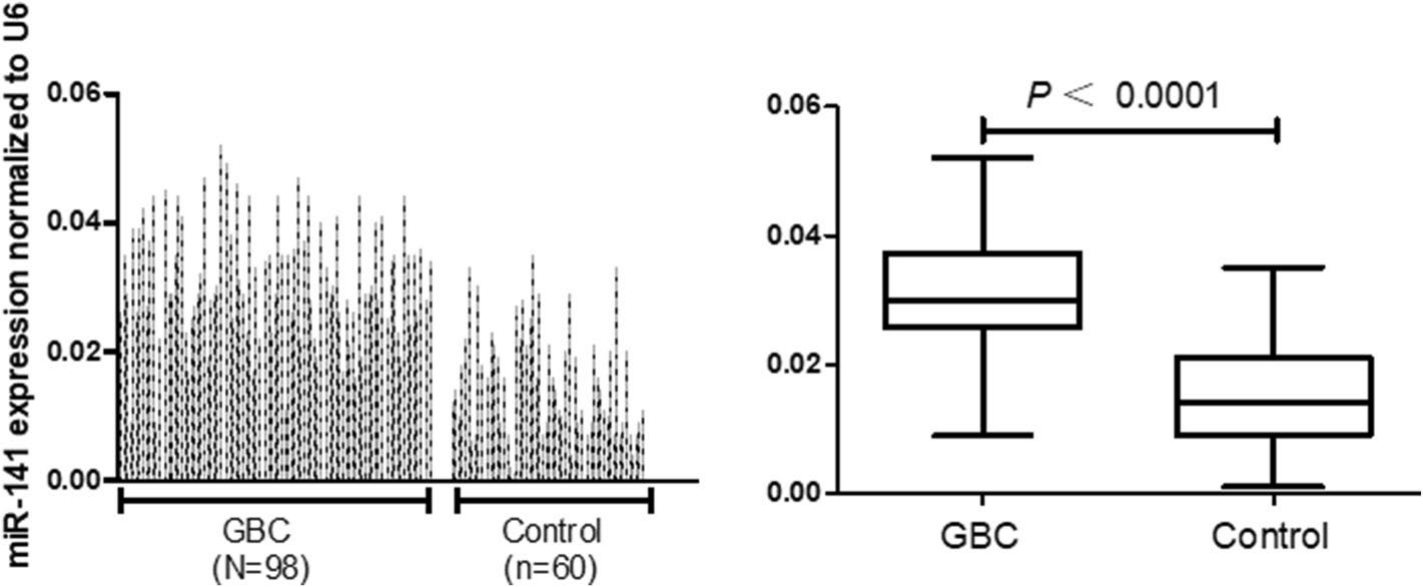
The expression levels of circulating miR-141 in plasma samples. The circulating miR-141 expression in plasma were significantly higher in gallbladder cancer (GBC) patients (n = 98) than in healthy controls (n = 60) (*P* < 0.0001; Mann–Whitney U-test). RNU6B (U6) was used as an internal control.

Real-time PCR was used to evaluate the expression level of miR-141 in the plasma of 98 GBC patients and 60 healthy volunteers. As shown in Fig. 2, the relative expression levels of miRNA-141 in plasma from GBC patients and healthy volunteers were 0.031 ± 0.009 and 0.015 ± 0.008, respectively, which presented a significant difference (*P* < 0.0001).

**Figure 3:**
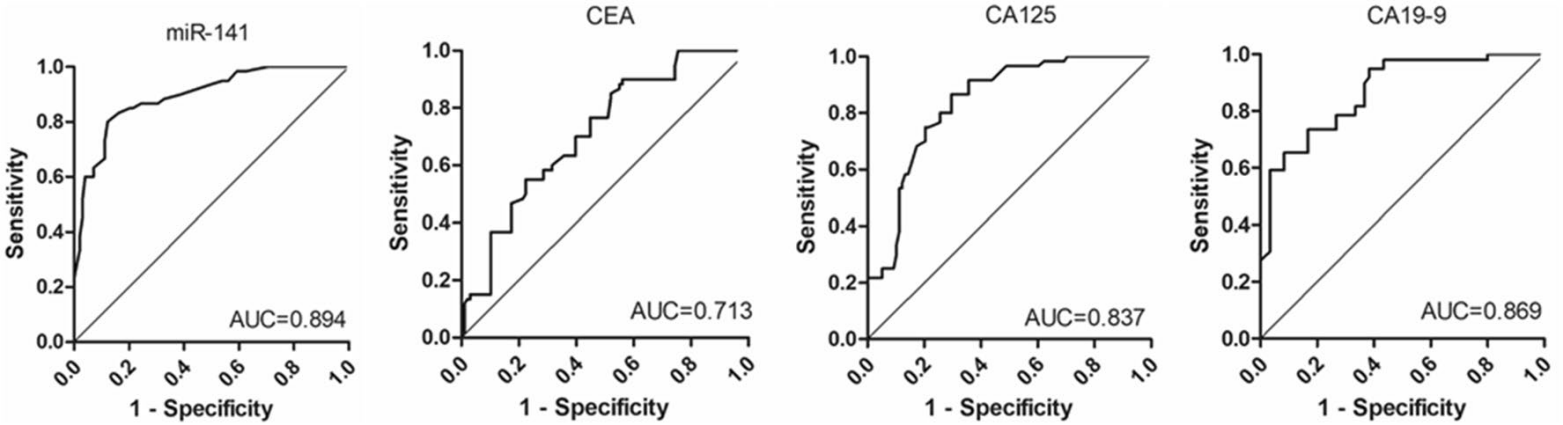
Comparison of the receiver operating characteristic (ROC) curves for miR-141 in the plasma and the conventional tumor biomarkers, carcinoembryonic antigen (CEA), carbohydrate antigen 125 (CA125) and carbohydrate antigen 19–9 (CA19–9) in the serum for gallbladder cancer patients.

Additionally, we evaluated the expression level of circulating miRNA-141 in peripheral blood as a new biomarker for clinical diagnosis in comparison with conventional tumor biomarkers, such as CEA, CA125 and CA19-9. Figure 3 exhibited the ROC curves that were obtained from ROC curve analysis. Our results showed that the diagnostic sensitivities of miR-141, CEA, CA125 and CA19-9 for GBC patients were 87.8, 43.9, 70.4 and 65.3% respectively, and the diagnostic specificities were 80.0, 90.0, 86.7, and 91.7% respectively. The AUC values of miR-141, CEA, CA125 and CA19-9 in GBC diagnosis were 0.894 (95% CI 0.843–0.945), 0.713 (95% CI 0.633–0.793), 0.837 (95% CI 0.776–0.899), and 0.869 (95% CI 0.813–0.924), respectively.

### Relationship between the expression level of plasma miR-141 and clinicopathological features in GBC patients

**Figure 4:**
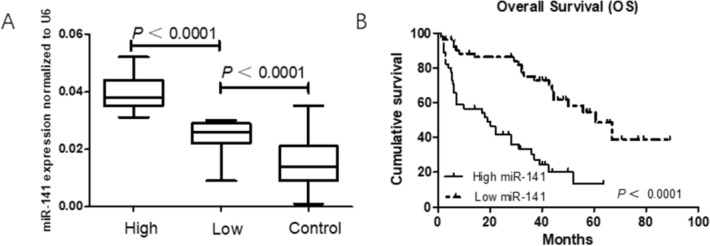
Survival analysis of 98 gallbladder cancer (GBC) patients stratified by high versus low miR-141 expression in plasma. (**A**) The levels of plasma miR-141 in the high-expression and low-expression and controls groups. (**B**) Kaplan–Meier analysis showing that the GBC patients with high miR-141 expression in the plasma displayed poorer OS (*P* < 0.0001; log-rank test).

The mean expression level (2^−ΔΔCt^ = 0.031) of miR-141 in GBC patients was used as the cutoff threshold to divide the GBC patients into high expression and low expression groups. According to this standard, the miRNA-141 expression level in plasma was upregulated in 45.92% (45/98) of the GBC patients and in 5.00% (3/60) of the healthy controls, which showed a significant difference (*P* < 0.0001) (Fig. 4A). Clinicopathological characteristics, including sex, age, differentiation, resection status, tumor invasion (T category), lymph node status (N category), pathologic tumor/node/metastasis (pTNM) stage, and adjuvant therapy, were compared between the two groups. The result showed that a high expression level of miRNA-141 was significantly correlated with tumor invasion (*P* = 0.008), lymph node status (*P* < 0.0001) and pTNM stage (*P* = 0.009). However, there was no significant differences in sex, age, resection status, differentiation, and adjuvant therapy between the two groups. The results are showed in Table 1.

**Table 1:** Associations between plasma miR-141 expression and the clinicopathological characteristics in patients with GBC.

	All	miR-141 expression		*χ*^2^ value	*P* value
		High	Low		
	98	45	53		
Sex					
Male	18	9	9	0.148	0.701
Female	80	36	44		
Age (y)					
< 60	51	22	29	0.331	0.565
≥ 60	47	23	24		
Differentiation					
Good	22	10	12	2.396	0.302
Moderate	42	16	26		
Poor	34	19	15		
Resection status					
R0	85	36	49	3.280	0.070
R1	13	9	4		
Tumor invasion					
T1–T2	23	5	18	7.075	0.008
T3–T4	75	40	35		
Lymph node status					
N0	62	18	44	19.883	< 0.0001
N1	24	17	7		
N2	12	10	2		
pTNM stage^a^					
I–II	20	4	16	6.797	0.009
III–IV	78	41	37		
Adjuvant therapy					
Yes	40	22	18	2.245	0.134
No	58	23	35		

^a^The clinicopathologic staging was performed according to the eighth edition of the criteria established by the International Union Against Cancer (UICC) in 2017. *pTNM* pathological tumor/node/metastasis. *GBC* gallbladder cancer.

### Elevated circulating miRNA-141 expression in peripheral blood was correlated with the poor survival of gallbladder cancer patients

During the follow-up period, 54 of 98 (55.10%) patients died. Based on the log-rank test analysis, we found that GBC patients with an elevated miRNA-141 expression in plasma presented a poorer 5-year OS rate than patients with a low miRNA-141 expression (14% vs 48%, *P* < 0.0001). The Kaplan–Meier cumulative survival curves for 98 GBC patients were shown in Fig. 4B. Additionally, we confirmed that survival time was correlated with age (*P* = 0.023), differentiation (*P* = 0.002), resection status (*P* ˂ 0.001), tumor invasion (*P* = 0.002), lymph node status (*P* ˂ 0.001), and pTNM stage (*P* = 0.003) according to the long-rank survival analysis. However, in GBC patients, survival time was not affected by the patient’s sex (*P* = 0.398) or adjuvant therapy (*P* = 0.354). The results of multivariate analysis demonstrated that R1 resection status, advanced pTNM stage and high circulating miR-141 expression in patients were independent prognostic factors for predicting poorer long-term survival in GBC patients, with hazard ratios of 4.142 (95% CI 1.927–8.903), 2.536 (95% 1.423–4.519) and 2.475 (95% CI 1.229–4.987), respectively. The results were shown in Table 2.

**Table 2:** Univariate and multivariate analysis for prognostic factors of GBC.

	Univariate			Multivariate		
	HR	95%CI	*P* value	HR	95%CI	*P* value
**Sex**						
Male						
Female	0.750	{0.385–1.460}	0.398			
**Age (y)**						
< 60						
≥ 60	1.873	{1.089–3.220}	0.023	1.464	{0.241–8.904}	0.679
**Differentiation**						
Good						
Moderate						
Poor	1.803	{1.235–2.632}	0.002	1.356	{0.887–2.074}	0.159
**Resection status**						
R0						
R1	5.828	{2.890–11.752}	< 0.001	4.142	{1.927–8.903}	< 0.001
**Tumor invasion**						
T1–T2						
T3–T4	3.790	{1.611–8.919}	0.002	2.162	{0.476–9.818}	0.318
**Lymph node status**						
N0						
N1						
N2	2.142	{1.528–3.002}	< 0.001	1.265	{0.793–2.018}	0.324
**pTNM stage**						
I–II						
III–IV	4.731	{1.701–13.158}	0.003	2.536	{1.423–4.519}	0.014
**Adjuvant therapy**						
Yes						
No	0.776	{0.454–1.327}	0.354			
**miR-141**						
Low expression						
High expression	3.843	{2.158–6.843}	< 0.001	2.475	{1.229–4.987}	0.011

*HR* hazard ratio, *CI* confidence interval, *pTNM* pathological tumor/node/metastasis, *GBC* gallbladder cancer.

### Effect of miR-141 on GBC cells proliferation viability

**Figure 5:**
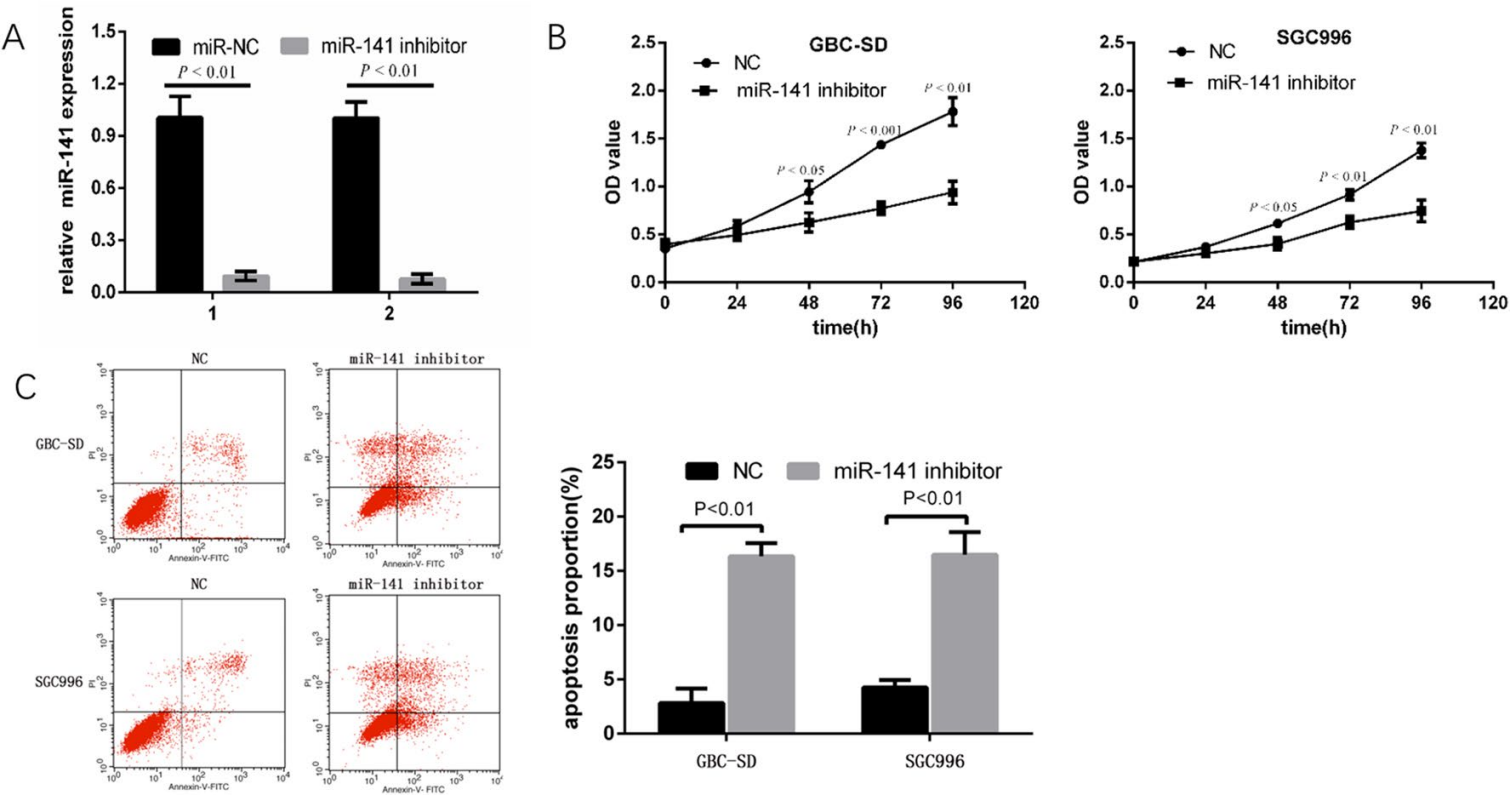
MiR-141 downregulated inhibited cell proliferation of GBC, but promoted cell apoptosis. (**A**) miR-141 was significantly decreased by using miR-141 inhibitor in GBC-SD cells and SGC996 cells. (**B**) The cell proliferation of these two cell lines was determined by MTT assays. (**C**) The apoptotic rates of GBC were tested.

To examine the proliferation viability of miR-141 in the GBC-SD and SGC996 cells. The miR-141 inhibitor was performed to downregulated the expression of miR-141 in GBC-SD and SGC996 cells. The cells were conducted 48 h after transfection with the miR-141 inhibitor or miR inhibitor control. qRT-PCR assay revealed that the expression of miR-141 was significantly decreased in miR-141 inhibitor transfected GBC cell lines compared with that control groups (both *P* < 0.05; Fig. 5A). More important, MTT assay demonstrated that the viability of GBC-SD and SGC996 cells in the miR-141 inhibitor transfection group was significantly decreased compared with that of control group (both *P* < 0.05; Fig. 5B). These results indicated that the downregulated of miR-141 reduced the proliferation of the GBC cells.

### Effect of miR-141 on the GBC cell apoptosis

To investigate the effect of miR-141 on the GBC cells apoptosis. The GBC-SD and SGC996 cells were transfected with miR-141 inhibitor or miR inhibitor control and cultured for 48 h following with analysis of apoptosis by flow cytometry. The results revealed that the proportion of apoptotic cells in the miR-141 inhibitor transfection group was significantly increased compared with that in control group (both *P* < 0.05; Fig. 5C). This assay indicated that reduced expression of miR-141 induced apoptosis in GBC cells.

## Discussion

GBC has emerged as a more aggressive disease and is usually diagnosed at an advanced tumor stage with metastasis due to the lack of typical early-stage clinical manifestation and the short of sufficiently effective and sensitive biomarkers, ultimately resulting in an extremely poor prognosis^22,23^. Previous studies have demonstrated that the most critical factors for predicting survival of GBC patients remain radical resection and tumor pTNM stage^24–26^. However, these factors are acquired postoperatively and are unconventional, which limits their wide clinical application to a certain extent. Although conventional tumor markers, such as CEA, CA125, and CA19-9, have been used clinically, their sensitivity and specificity in diagnosis and prognosis are not enough. Therefore, the discovery of specific and sensitive tumor markers, especially the blood-based biomarker for early-stage GBC is urgent. In this study, we hypothesized that the expression of circulating miRNA-141 in plasma maybe have a feasible predictive value for the diagnosis, therapy and survival of GBC patients.

MiRNAs, one class of small noncoding RNA molecules, play a vital role in the development and progression of tumors by regulating the expression of many key genes at the posttranscriptional level. Further elucidation of the correlation between miRNAs and tumorigenesis may contribute to a deeper understanding of cancers and lead to the identification of potential diagnostic and prognostic parameters as well as therapeutic targets in clinical applications^27,28^. Previous studies have confirmed that miRNAs can suppress and even eliminate the development of various tumors in vitro and vivo and present a dual tumorigenic capacity^6,29,30^. In addition, accumulating research have demonstrated that the circulating miRNAs in plasma present the potential to be served as tumor biomarkers for the early-stage diagnosis, therapy and prognosis of several human malignances due to their remarkably stable expression, even when degraded by ribonuclease in peripheral blood^9–12,29,31,32^.

MiRNA-141 is a vital carcinogen associated with a high incidence of lymph node metastasis, distal metastasis and poor prognosis by regulating the cell cycle, cell differentiation, apoptosis and invasion, as reported in previous studies. For example, Wang et al. demonstrated that elevated miR-141 expression was associated with diagnosis and favorable prognosis of patients with bladder cancer^33^. Mak CS et al.^34^ confirmed that elevated expression of miR-141 promotes, while the downregulated the expression of miR-141 inhibits cell proliferation, anchorage-independent capacity, anoikis resistance, tumor growth and peritoneal metastases of ovarian cancer cells through targeting the KLF12/Sp1/survivin axis. Additionally, Kim et al.^19^ reported that the overexpression of miRNA 141 was significantly associated with shorter disease-free survival and a greater risk of angiolymphatic invasion in biliary tract cancer patients who had undergone R0 resection; moreover, among the patients who did not undergo R0 resection, miRNA 141 overexpression was significantly associated with reduced OS. However, Li et al.^15^ found that miR-141 was significantly decreased and was correlated with advanced TNM stage and lymph node metastases in gastric cardia adenocarcinoma patients, and increased miR-141 expression in gastric cancer cells could suppress cell proliferation by targeting MACC1. Similarly, Han et al.^35^ suggested that miR-141 knockdown causes ERBB4 overexpression, which is involved in trastuzumab resistance in breast cancer cells and is associated with poor outcomes in breast cancer patients. The above research shows that miR-141 plays a dual role in tumorigenicity, modulates cellular motility and presents a certain function of tumor stem cells. This phenomenon strongly reveals that miR-141 is a critical oncogene or tumor suppressor gene and presents potential options for the diagnostic, therapeutic and prognostic target of cancer. Nevertheless, we believe that miRNA-141 acts as an oncogene and may play a vital role in the diagnosis, therapy and prognosis of GBC patients based on a recent study.

In the present study, we first confirmed the expression level of miRNA-141 in GBC cells and tissues. The results showed that aberrant elevated miRNA-141 expression was detected in four GBC cell lines and GBC tissues, which was consistent with that in previous studies. Then, we detected the level of plasma miRNA-141 in GBC patients and healthy individuals. The expectant result that the expression of circulating miRNA-141 in GBC patients was statistically higher than in controls was obtained, which provided strong evidence that GBC-related miRNA could be released into the circulation and remained at a stable level in blood. Additionally, in this study, we also discovered that the expression of circulating miR-141 in plasma had a clinically satisfactory degree of specificity and sensitivity with an AUC of 0.894, which presented a greater advantage than the AUC values obtained from conventional tumor biomarkers for GBC, such as CEA, CA125 and CA19-9. These results revealed that circulating miR-141 levels in plasma may be more valuable than CEA, CA125 and CA19-9 levels for GBC diagnosis. Moreover, we found that plasma miRNA-141 expression was upregulated in 45.92% (45/98) of GBC patients and in 5.00% (3/60) of healthy controls according to the cutoff value, which presented an adequate tumor marker specificity and sensitivity for the diagnosis of GBC patients. More important, we explored the prognostic value of miR-141 expression levels. Our findings demonstrated that plasma miRNA-141 expression was significantly correlated with tumor invasion, lymph node status and pTNM stage and predicted the OS of patients with GBC. This promising result provides evidence that the plasma miRNA-141 expression level may be helpful for establishing an accurate pTNM stage and determining tumor invasion and the lymph node status preoperatively, and a blood-based candidate tumor marker for GBC patients’ diagnosis and prognosis is available. In addition, we explored the tumor biological roles of miR-141 in GBC-SD and SGC996 cell lines. The results revealed that miR-141 acting as an oncogene could inhibit the apoptosis and promote the proliferation of GBC cells. Importantly, these oncogenic functions in GBC cells could be reversed by transfection with miR-141 inhibitor. These results indicated that miR-141 could be used as potential therapeutic targets for the treatment of GBC. Due to the limited number of samples and lack of relevant research on downstream target genes of miR-141 in the current study, a continuing study with larger sample sizes and mechanism involved are needed to further confirm this important finding.

These data strongly suggest that miRNA-141 was overexpressed in gallbladder cancer cell lines and tissues and was stably expressed in plasma. And miR-141 acting as an oncogene could inhibit the apoptosis and promote the proliferation of GBC cells. Additionally, the circulating miR-141 levels in plasma were more valuable than the levels of CEA, CA125 and CA19-9 for GBC diagnosis. Meanwhile, the high expression level of circulating miRNA-141 was significantly associated with tumor invasion, lymph node metastasis and advanced pTNM stage. More importantly, elevated circulating miRNA-141 expression might be a potential novel biomarker for diagnosis and predicting the poor prognosis of GBC patients.

## Data Availability

The datasets generated and/or analyzed during the current study are not publicly available due to restrictions (institutional policy to protect the privacy of research participants) but are available from the corresponding author on reasonable request.
